# Basal glucosuria in cats

**DOI:** 10.1111/jpn.13018

**Published:** 2018-10-29

**Authors:** Florian Karl Zeugswetter, Theresa Polsterer, Herbert Krempl, Ilse Schwendenwein

**Affiliations:** ^1^ Clinical Department for Companion Animals and Horses University of Veterinary Medicine Vienna Austria; ^2^ Tierambulatorium Heiligenstadt Vienna Austria; ^3^ Department of Pathobiology University of Veterinary Medicine Vienna Austria

**Keywords:** cats, glucose, glucose‐to‐creatinine ratio, urine

## Abstract

Objective of this study was to demonstrate the ubiquitous presence of glucose in urine of euglycemic cats by a highly sensitive glucose assay. The local electronic database was searched for results of quantitative urine glucose measurements in cats. A total of 325 feline urine glucose measurements were identified, of which 303 (93%) had been submitted by one of the co‐authors working in a near‐by small animal practice. After the exclusion of patients with kidney disease (*n* = 60), hyperthyroidism (*n* = 15), diabetes mellitus (*n* = 11), multiple diseases (*n* = 9) or steroid treatment (*n* = 3), as well as serial measurements (*n* = 87) and outliers (*n* = 8), the final study population consisted of 132 cats. Urine creatinine concentration was unavailable in five patients. Whereas all but one cat had glucose concentrations above the detection limit of the assay (0.11 mmol/L, Gluco‐quant Enzyme Kit/Roche Diagnostics), no positive glucose dipstick test result (Combur 9‐Test, Roche Diagnostics) was observed. The median (range) of urinary glucose concentration and the glucose‐to‐creatinine ratio (UGCR) was 0.389 (<0.11–1.665) mmol/L and 0.0258 (0.007–0.517) respectively. The UGCR was not affected by age, gender, breed or leukocyturia, whereas cats with hematuria had slightly higher values. Data show that so‐called “basal glucosuria” is present in the majority of cats and by no means diagnostic for diabetes mellitus or renal glucosuria. This has to be considered when using bio‐analytical methods with a low limit of quantification.

## 1 INTRODUCTION

Urine glucose measurement is an integral part of routine urinalysis (Reine & Langston, 2005) and essential for the diagnostic workup of cats with polyuria, polydipsia and weight loss. It is furthermore a widely used (Aptekam, Armstrong, Coradini, & Rand, 2014), but imperfect method to adjust insulin doses in cats with diabetes mellitus (DM, Roomp & Rand, 2013). Up to blood glucose concentrations of about 14.98 mmol/L (270 mg/dl) tubular reabsorption of filtered glucose is linear and almost complete (Eggleton & Shuster, 1954). Above this “renal threshold,” which has a wide interindividual variation, linearity is lost and although the proximal convoluted tubules continue to reabsorb glucose at a maximum rate, reabsorption declines below 50% (Eggleton & Shuster, 1954). At this point, urinary glucose concentrations increase and may become detectable depending on the analytical method employed. Rarely, idiopathic or acquired defects in the absorptive mechanisms of the proximal convoluted tubules cause so‐called “renal glucosuria.” Renal glucosuria in association with variable losses of amino acids, uric acid, ions and electrolytes is termed Fanconi syndrome (Reinert & Feldman, 2016). Taken together, “glucosuria” indicates the presence of elevated plasma glucose concentrations, an impaired renal glucose absorptive capacity, or both.

The current methodology to measure urinary glucose is the use of semiquantitative dry reagent strips employing an enzymatic oxidase–peroxidase chromogen coupled reaction. The term “glucosuria” is traditionally confined to a positive dipstick test result. The fact that various commercially available test strips have different lower limits of detection for glucose, ranging from ~1.1 mmol/L (20 mg/dl) to ~5.5 mmol/L (100 mg/dl), is widely ignored. The most sensitive test strips currently validated for the use in cats give positive colour reactions at glucose concentrations of about 2.78 mmol/L (50 mg/dl, Defontis, Bauer, Failing, & Moritz, 2013). Several studies in healthy and sick humans have shown that glucose is virtually present in all urine samples (Apthorp, 1957; Brunner, Kurz, Adelsmayr, & Holzinger, 2015; Fine, 1965; Wolf, Rave, Heinemann, & Roggen, 2009). In 1965, Fine proposed to replace the term “glucosuria,” by the terms “normoglucuria” and “hyperglucuria.” In modern literature, the excretion of glucose at euglycaemic blood glucose concentrations and a normal tubular function is named basal glucosuria (Rave et al., 2006; Wolf et al., 2009). As basal glucosuria is independent of blood glucose concentrations, urinary flow rates, renal threshold and maximal rate of tubular absorption, it is assumed to be the result of distal tubular leakage (Rave et al., 2006).

The primary objective of this retrospective study was to determine whether glucose is present in feline urine samples and to define approximate reference limits of glucose and the urine‐to‐glucose ratio (UGCR). The subsequent aim was to investigate possible influencing factors.

## 2 MATERIALS AND METHODS

In a retrospective study, the electronic database of the teaching hospital (TIS VetWare (Agfa Health Care)) at the University of Veterinary Medicine Vienna/Austria was searched for results of quantitative urine glucose measurements in feline urine. The observation period was from August 2006 to August 2016. The search terms applied were “cats” and “glucose urine.” Data collected included breed, gender, age, weight, method of urine collection and results of urinalysis as well as simultaneous blood work. In case of multiple consignments from the same animal, only the results of the first urine sample were included. All measurements were performed at the local university laboratory. Standard techniques for urinalysis included refractometry (RHC‐200ATC), sediment microscopy, dipstick analysis (Combur 9‐Test, Roche Diagnostics [lowest point estimate for glucose 2.78 mmol/L or 50 mg/dl]) as well as quantitative measurements of glucose, creatinine and protein on a Roche/Hitachi Cobas® c 502 analyzer. Unstained urine sediment was examined for red and white blood cells by light microscopy at 400x. Hematuria and leukocyturia were defined as >8 and 5 cells per high power field (Welles, Whatley, Hall, & Wright, 2006) respectively. Glucose, fructosamine and creatinine concentrations were measured with the hexokinase method (Gluco‐quant Enzyme Kit/Roche Diagnostics), a colorimetric assay (Fructosamine; Roche Diagnostics) and Creatinine plus version 2 (Roche Diagnostics) respectively. For validation of the glucose determinations in feline urine, a dilution/recovery study was performed using feline urine with high (53 mmol/L) and unmeasurable glucose concentrations. The average recovery was 108%. The lower limit of detection was 0.11 mmol/L. The intra‐assay coefficient of variation (mean ± standard deviation [*SD*]) was calculated by assessing 10 replicates of urine samples with low (0.61 ± 0 mmol/L), medium (5.76 ± 0.07 mmol/L) and high (39.3 ± 0.36 mmol/L) glucose concentrations. The coefficient of variations (CV%) were 0%, 1.2% and 0.9% respectively. From day to day precision (cumulative quality control) was determined by measuring two commercially available aqueous control solution (Lyphocheck®1 and 2, Biorad, Vienna, Austria; target values: 1.2 and 17 mmol/L) over 10 consecutive days and revealed a CV% of 5% and 1.8% respectively.

Concentrations in excess were automatically diluted 1:20 and reanalysed. Internal quality control samples for all analytes were routinely run every day. To determine an upper reference limit, cats with suspected (glucose >7 mmol/L [126 mg/dl (Gilor, Niessen, Furrow, & DiBartola, 2016) and glucosuria [dipstick] and/or fructosamine >340 µmol/L) or treated diabetes mellitus (DM), and with diseases/treatments possibly effecting fractional glucose clearance such as hyperthyroidism (HT, thyroxine >45.57 nmol/L, da Silva Teixeira et al. 2016), acute or chronic kidney disease ≥stage 2 (KD, creatinine >141.44 µmol/L (>1.6 mg/dl), urine specific gravity (USG <1.035, Brown, 2016) or current glucocorticoid treatment (Lowe, Graves, Campbell, & Schaeffer, 2009) were excluded. To assess associations between renal function and glucosuria, cats with CKD ≥stage 2 were further subclassified according to the International Renal Interest Society (IRIS) into stages 2–4 (Brown, 2016). The urinary glucose‐to‐creatinine ratio (UGCR) was calculated by urine glucose (mmol/L) divided by urine creatinine (mmol/L).

Statistical analysis was performed with the laboratory software package IBM SPSS Statistics 24. Associations between pairwise variables were examined using Spearman's rank order correlation (*r*_SP_), and for group‐wise comparisons, the Mann–Whitney *U* test was employed. Correlation was classified as excellent (*r*_SP_ = 0.93–0.99), good (*r*_SP_ = 0.8–0.92), fair (*r*_SP_ = 0.59–0.79) or poor (*r*_SP_ < 0.59) respectively (Papasouliotis et al., 2008). Outliers were defined as data higher than three interquartile ranges (3 IQR) above the third quartile (Q3). The upper reference limits were determined by calculating the 0.975‐fractiles. The level of significance was set at *p* < 0.05. According to the distribution of the data (Shapiro–Wilk test), results are presented as median and range.

## 3 RESULTS

The search terms identified 325 feline urine glucose measurements; 303 (93%) of the samples had been submitted by one of the co‐authors (H.K.) working in a near‐by small animal practice. Urine had been routinely collected by manually expressing the bladder (private practice) or cystocentesis (university). Blood and urine samples had been stored at 4°C and submitted to the laboratory within 5 hr. KD, HT and DM were diagnosed in 67, 22 and 15 cats respectively. Nine of these cats (five with HT and KD, two with DM and KD, two with HT and DM) were allocated to more than one category. For the results of cats with DM, HT, KD as well as from the final study group see Table 1.

**Table 1:** Results of urine and blood analysis in the study groups

	Reference population	Diabetes mellitus	Hyperthyroidism	Kidney disease	Reference range
Number of cats	132	15	22	67	
Age (years, mean ±SD)	9.1 (±5.1)	12.2 (±2.8)**	13.8 (±3.7)***	13.4 (±3.7)***	
Urinalysis					
Glucose (mmol/L)	0.389 (n.d.–1.665)	48.008 (0.278–401.154)***	0.278 (0.056–44.4)	0.167 (n.d.–285.992)***	*n*.a.
Creatinine (mmol/L)	24.1 (2.1–80)	6.9 (2–39.2)**	9.3 (2.7–81.2)***	8 (1.9–24.2)***	*n*.a.
Glucose‐to‐creatinine ratio	0.0258 (0.007–0.517)	16.666 (0.022–214.459)***	0.048 (0.001–4.788)**	0.0279 (0.005–167.85)	*n*.a.
Protein (mg/L)	432 (57–3,365)	859 (130–2,773)	317 (130–3,200)	228 (63–7,230)***	20–630
Protein‐to‐creatinine ratio	0.16 (0.04–2.12)	0.32 (0.17–2.21)**	0.39 (0.08–0.81)***	0.24 (0.05–4.93)**	<0.33
Specific gravity	1.038 (1.003–1.061)	1.026 (1.008–1.049)	1.024 (1.009–1.055)**	1.014 (1.006–1.031)***	1.020–1.040
Blood analysis					
Glucose (mmol/L)	5.55 (1.277–14.042)	19.259 (2.775–33.189)***	5.883 (3.106–29.97)	5.328 (2.109–32.357)	3.053–5.55
Fructosamine (µmol/L)	271 (208–328)	573 (256–884)***	234 (114–267)**	254 (153–763)	<340
Creatinine (µmol/L)	123.76 (53.04–13,613.91)	106.08 (53.04–185.64)	97.24 (44.2–265.21)	203.32 (141.44–1,264.15)***	<141.44
Urea (mmol/L)	9.13 (3.06–25.8)	11.46 (4,07–24)	11.24 (3.66–28.46)	14.86 (4.29–102.27)***	3.34–10.86
Phosphorus (mmol/L)	1.31 (0.8–2.95)	1.26 (0.81–1.57)	1.45 (0.99–2.26)	1.46 (0.81–7.77)*	0.8–1.6
Thyroxine (nmol/L)	18.85 (12–42)	15.3 (15–34)	130 (34–310)***	20.2 (12–130)	19–45.57

Note

*n*.a.: not available, n.d.: not detectable, difference to reference population (Mann–Whitney *U* test, age: *t* test)

*** *p* < 0.001;

** *p* < 0.01;

* *p* < 0.05

**Figure 1:**
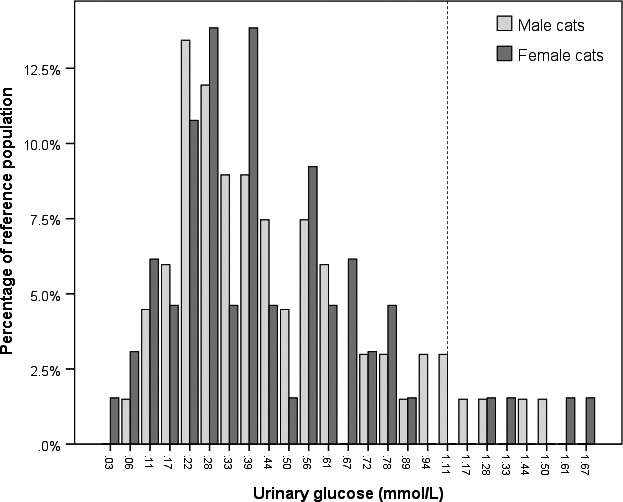
Distribution of urinary glucose concentrations in the 132 cats grouped according to gender. None of these cats had a positive glucose dipstick test result (lowest point estimate 2.78 mmol/L). The vertical dotted line represents the lowest point estimate of a sensitive modern urinary dipstick test (1.11 mmol/L)

After the exclusion of patients with KD, HT, DM or glucocorticoid treatment (*n* = 3) as well as serial measurements (*n* = 87) and outliers (*n* = 8), the final study population consisted of 132 cats; 128 (96.7%) of the urine samples had been collected by manually expressing the bladder. Urinary creatinine concentrations were unavailable in five cats. The upper reference limits for glucose and the UGCR were 1.48 mmol/L (26.675 mg/dl) and 0.081 respectively. For distribution of urinary glucose concentrations separated according to gender see Figure 1. None of these cats had a positive glucose dipstick test result.

Common breeds included 116 domestic shorthair mixed breeds (88%) and eight Persian cats (6%). The cats consisted of 67 (51%) males (9 intact) and 65 (49%) females (10 intact). Urinary glucose was below the detection limit of the assay in one cat (0.76%).

Gender and breed had no effect on the urinary glucose concentrations (*Z* = −0.377, *p* = 0.706; *Z* = −1.134, *p* = 0.257) or the UGCRs (*Z* = −1.268, *p* = 0.205; *Z* = −0.947, *p* = 0.344). Age correlated negatively with glucose _(urine)_ (see Table 2), Crea_(urine)_ (*r*_SP_ = −0.241, *p* = 0.012) and USG (*r*_SP_ = −0.313, *p* = 0.001). Leukocyturia and hematuria were detected in 39 (26%) and 59 (45%) patients respectively. Urinary glucose concentrations (*Z* = −1.445, *p* = 0.149) and the UGCRs (*Z* = −0.763, *p* = 0.445) were comparable in cats with and without leukocyturia. Cats with hematuria had slightly higher urinary glucose concentrations 0.44 mmol/L [0.03–1.67 mmol/L] versus 0.41 mmol/L [0.06–1.44 mmol/L], *Z* = −2.178, *p* = 0.029) and UGCRs (0.03 [0.007–0.517] vs. 0.025 [0.014–0.16], *Z* = −2.388, *p* = 0.017) than cats without hematuria.

**Table 2:** Correlations between urinary glucose, glucose‐to‐creatinine ratio and other parameters

	Glucose (urine)	UGCR
Age		
Correlation	−0.223	0.005
Significance (2‐sided)	0.019	0.96
*n*	111	106
Glucose (plasma)		
Correlation	0.404	0.235
Significance (2‐sided)	<0.001	0.028
*n*	89	87
Glucose (urine)		
Correlation	1	0.325
Significance (2‐sided)		<0.001
*n*	132	127
Fructosamine (plasma)		
Correlation	0.270	0.061
Significance (2‐sided)	0.263	0.81
*n*	19	18
Creatinine (plasma)		
Correlation	−0.012	−0.470
Significance (2‐sided)	0.894	<0.001
*n*	118	114
Creatinine (urine)		
Correlation	0.667	−0.381
Significance (2‐sided)	<0.001	<0.001
*n*	128	127
Urea (plasma)		
Correlation	−0.174	−0.119
Significance (2‐sided)	0.062	0.209
*n*	116	113
Phosphorus (plasma)		
Correlation	−0.174	0.081
Significance (2‐sided)	0.064	0.399
*n*	114	111
Thyroxine (serum)		
Correlation	0.141	−0.069
Significance (2‐sided)	0.414	0.698
*n*	36	34
Protein (urine)		
Correlation	0.569	0.04
Significance (2‐sided)	<0.001	0.657
*n*	128	126
Specific gravity (urine)		
Correlation	0.676	−0.145
Significance (2‐sided)	<0.001	0.109
*n*	127	124
UPCR		
Correlation	−0.004	0.390
Significance (2‐sided)	0.968	<0.001
*n*	127	126

Note

UGCR: urinary glucose‐to‐creatinine ratio, UPC: urinary protein‐to‐creatinine ratio, shaded fields depict significant correlations

**Figure 2:**
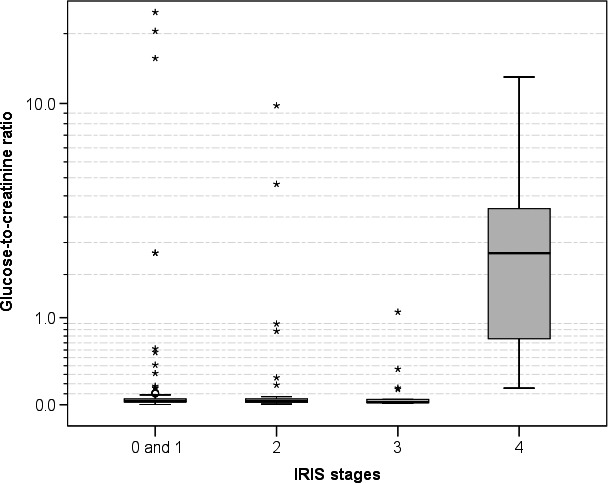
Box plots of glucose‐to‐creatinine ratios in cats grouped according to IRIS stages 0–4

Of the 67 cats with renal insufficiency, 42, 18 and 7 were assigned IRIS stages 2, 3 and 4 of 4 respectively. Cats with IRIS stage 4 had significantly higher glucose concentrations (Figure 2) and UGCRs than cats with stages 0 and 1 (glucose: *Z* = −2.469, *p* = 0.014; UGCR: *Z* = −4.405, *p* < 0.001), stage 2 (*Z* = −3.543, *p* = 0.011; *Z* = −3.406, *p* < 0.001) and stage 3 (*Z* = −3.304, *p* = 0.001; *Z* = −3.461, <0.001) respectively.

## 4 DISCUSSION

Data of this study confirm that, as has already been shown in humans (Fine, 1965; Rave et al., 2006; Wolf et al., 2009; Brunner et al., 2015) and a small cohort of non‐diabetic obese cats (Hoenig, Clark, Schaeffer, & Reiche, 2018), glucosuria is present in euglycemic cats. With the use of a highly sensitive assay, the mean (±*SD*) urinary glucose concentration was 0.46 (±0.32) mmol/L [8.25 (±5.76) mg/dl], which is very close to the 0.7 (±0.3) mmol/L [12.6 (±5.4) mg/dl] reported by Hoenig et al. (2018). Accordingly, the presence of small amounts of glucose in feline urine is by no means diagnostic for diabetes mellitus or renal glucosuria. We therefore propose to implement the term “basal glucosuria,” which was first used by Lazarus (1904) and is now standard in modern human literature (Rave et al., 2006; Wolf et al., 2009). The terms “normoglucuria” and “hyperglucuria” (Fine, 1965) would also be applicable.

Comparable to the results in normal humans (Fine, 1965; Gupta, Goyal, Ghosh, Punjabi, & Singh, 1982), no significant effect of gender was observed. Our data also suggest no influence of breed. Older cats had lower glucose concentrations, but this effect was likely caused by dilution as no difference was detected in the UGCRs.

Noteworthy, seven per cent of the non‐diabetic, non‐azotemic cats in this study had urinary glucose concentrations higher than 1.11 mmol/L (20 mg/dl), which is high enough to generate positive colour reactions if modern, highly sensitive dipsticks are used (e.g., Medi‐Test Glucose, Marchery‐Nagel, Dueren, Germany). In humans, with the use of these test strips a yellow to weakly green test field is considered normal. Consequently, the statement in the ISFM‐guidelines on DM in cats (Sparkes et al., 2015) that “persistent glucosuria suggests inadequate control” does not apply for quantitative laboratory methods and likely only for traditional dipsticks with a lowest point estimate of 2.78 mmol/L (50 mg/dl) or higher.

Although the urinary glucose concentrations in cats with hyperthyroidism were comparable to the concentrations observed in the reference population, the urine was more diluted resulting in higher UGCRs. Increased glucose losses were also observed in a recent study using Wistar rats with alloxan‐induced DM. The intraperitoneal application of triiodothyronine at supraphysiological doses resulted in a reduction of kidney sodium‐glucose cotransporter 2 (SGLT‐2) expression, reduced renal glucose reabsorption and accordingly increased glucosuria (da Silva Teixeira et al., 2016).

In accordance with the results in human patients with non‐diabetic advanced CKD where fractional glucose excretion increases significantly with the decline of renal function (Hung et al., 2016), all seven cats with IRIS stage 4 (of 4) KD had significantly elevated UGCRs and five of these had a positive glucose dipstick test result. It is unknown, whether hyperglucuria in patients with CKD indicates a proximal tubulopathy, a dysfunction of the SGLTs‐2 or both. In a human study, the percentage of positive glucose dipstick results increased from 3.4% to 56.3% in patients with progression from stage 3 to stage 5 (of 5) CKD. Despite the fact that glucosuria was associated with an increased fractional excretion of electrolytes and a higher urinary protein‐to‐creatinine ratios, glucosuric patients with stage five CKD had a 0.77‐ and 0.63‐fold lower risk for end‐stage renal failure and rapid decline of renal function respectively. The authors speculated that damage of the proximal tubules in this subset of patients might reduce protein reabsorption and hence tubulointerstitial protein accumulation, thereby limiting cytokine activation, inflammation and fibrogenesis (Hung et al., 2016). Prospective studies are now needed to clarify whether cats with CKD and concomitant pathological glucosuria also have a more favourable outcome.

Although the upper reference limit of 1.48 mmol/L [26.675 mg/dl] for urinary glucose in this study is very close to the currently suggested cut‐off for humans (1.4 mmol/L [25 mg/dl], Cowart & Stachura, 1990), it has to be used with caution.

It is possible that cats with blood glucose concentrations originally above the individual renal threshold were unintentionally included. Blood samples had been stored for up to 5 hr and although cooled, had neither been separated from blood cells nor fluorinated. In contrast to urinary glucose, which is stable for at least 5 days if stored at 4°C (Fletcher, Behrend, Welles, Lee, & Hosgood, 2011), blood glucose may drop by as much as 7% per hour if no anti‐glycolytic agents are added (van den Berg, Thelen, Salden, van Thiel, & Boonen, 2015). Non‐standardized preanalytical specimen handling was also the reason why fractional glucose excretion was not calculated. Prospective studies with healthy cats, perhaps including euglycemic‐clamp techniques or continuous glucose monitoring systems, are now necessary to more accurately describe feline basal glucosuria.

Although all cats with acute KD and IRIS stages 2–4 CKD were excluded for the calculation of the above‐named reference intervals, specific urine metabolic assays (e.g., amino acids, organic acids) were not applied and the incidental inclusion of patients with defects in the absorptive mechanisms of the proximal convoluted tubules is possible. To our knowledge, there are only three published reports describing acquired renal glucosuria in cats (Hoenig et al., 2018; Lowe et al., 2009; Reinert & Feldman, 2016), whereas hereditary forms have not been described. Triggering events were the application of dexamethasone (Lowe et al., 2009), various glucocorticoids in combination with chlorambucil (Reinert & Feldman, 2016) and velagliflozin (Hoenig et al., 2018), a SGLT‐2 inhibitor. SGLTs‐2 are important membrane proteins located at the luminal membrane of the proximal renal tubular cells and enable the active re‐uptake of glucose which is freely filtered at the glomerulus. Mutations of the gene encoding these transporters cause familiar renal glucosuria, and selective inhibition is a new therapeutic option for the treatment of type‐2 diabetes mellitus in humans (Lajara, 2014). In rats, dexamethasone increases urinary glucose excretion in a dose‐dependent manner regardless of plasma glucose or insulin concentrations. A selective action on glucose transport and metabolism in tubular cells is assumed (Yamanouchi et al., 1998). None of the cats in the final study population received glucocorticoids or chlorambucil and SGLT‐2 inhibitors are not yet used in veterinary clinical practice.

The measurement of the absolute glucose concentrations does not incorporate urine flow rate, which is modulated by the hydration status and the renal capacity for reabsorption of free water. To account for dilutional effects the incorporation of urinary creatinine concentrations, which is already standard practice for other biomolecules such as proteins (Vilhena et al., 2015; Welles et al., 2006) and steroids (AlCauvin et al., 2003) is advisable. The calculation of the UGCR also eliminated the effects of age, which was negatively correlated with urinary glucose concentration, USG and creatinine concentrations in the present study. In a recent veterinary study, the UGCR was calculated to investigate the effects of a SGLT‐2 inhibitor in obese cats (Hoenig et al., 2018). Within 35 days, the UGCR increased from mean (±*SD*) 0.016 ± 0.006 to 26.31 ± 2.94. The results of the present study suggest that a UGCR above 0.081 is abnormal. In human type‐2 diabetics, the UGCR of the first morning urine samples shows good linear relationship with the overnight eight hours UGCR (Kim et al., 2017) and correlates fairly with various glycemic indices such as basal and stimulated blood glucose, glycated albumin (%) and glycated haemoglobin (Kim et al., 2017). It has already been shown that urinary dipstick glucose determination is a useful indicator of clinical control in diabetic cats treated with porcine zinc insulin (Martin & Rand, 2007). Studies are now needed to clarify whether quantitative glucose measurements and the integration of creatinine can actually improve diagnostic accuracy and treatment surveillance of insulin‐treated diabetic cats.

In conclusion, this study shows that feline urine is virtually never free of glucose and that basal glucose excretion is not effected by age, gender or breed. The introduction of the concept of “basal glucosuria,” which is well established in human medicine, seems justified.
